# Prevalence and factors associated with neonatal acute respiratory distress syndrome among neonates admitted to the neonatal intensive care units of Gurage zone public hospital, South West Ethiopia

**DOI:** 10.4314/ahs.v23i3.20

**Published:** 2023-09

**Authors:** Bogale Chekole, Terefe Tamene Fetene, Tenaw Shegaw Geze, Zewudie Bitew Tefera, Gebre Eyesus Fisha Alebel, Amare Kassaw, Walle Belete Gelaw, Zeleke Fentahun Tamene, Yemsirach Mira, Tesfu Mulatu, Derartu Deressa

**Affiliations:** 1 Department of Nursing, College of Medicine and Health Science, Wolkite University Southwest Ethiopia; 2 Department of Midwifery, College of Medicine and Health Science, Wolkite University Southwest Ethiopia; 3 Department of Pediatric Nursing, College of Medicine and Health Science, Debre Tabor University, Northwest Ethiopia; 4 Department of Pediatric Nursing, College of Medicine and Health Science, Wolaita Sodo University, Southwest Ethiopia

**Keywords:** Respiratory distress syndrome, neonate

## Abstract

**Background:**

Respiratory distress syndrome (RDS) is the leading cause of respiratory failure and death of a neonate in today's world, especially in developing countries like Ethiopia.

**Methods:**

We used an institutional-based cross-sectional study in the selected hospitals of the Gurage zone admitted from June 2019 to June 2021. The data were collected using a structured questionnaire. Data were entered into Epi data 3.1 and exported to SPSS version 25 for analysis.

**Result:**

The prevalence of respiratory distress syndrome (RDS) in the study area was 45.1%. The odds of RDS in neonates from mothers with gestational age between 35 &37 were 3.99 times higher compared to term gestation. The odds of RDS among neonates with jaundice and sepsis are 4.33- and 1.92-times higher odds compared to their counterparts. The odds of RDS in neonates born via Caesarean section were 1.7 times higher compared with those delivered via spontaneous and instrumental delivery. RDS was also higher in neonates born to mothers <20 years of age and >=35 years old.

**Conclusion:**

the prevalence of RDS in the study area was high. Thus, healthcare providers should act on those factors with appropriate follow-up for early detection of the problem and prevent the risk.

## Introduction

According to various studies, Respiratory distress syndrome(RDS) is the leading cause of neonatal morbidity and mortality^1^, ^2^. The burden and factors associated with neonatal RDS were not well studied in low-income countries, particularly in Sub-Saharan Africa^3^, which is the leading cause of neonatal morbidity and mortality in various studies^3^. Despite some progress in the care of neonatal patients in Sub-Saharan Africa, it is still slow 4. One form of respiratory distress is acute respiratory distress syndrome (ARDS), which causes fluid to build up in the lungs, inhibiting breathing and the transfer of oxygen into the bloodstream. ARDS usually develops in patients who are already dealing with another disease. It is one of the severe health problems of neonates in developing countries like Ethiopia. ARDS, sepsis, Jaundice and other neonatal problems diagnosis is based on the national NICU management protocol^5^.

Due to the evidence of high neonatal morbidity and mortality by Neonatal respiratory syndrome, the world health organization is working with ministries of health of the nations and its partners to strengthen and invest in care, particularly around the time of birth and the first week of life. However, regional and national data on the impact of these efforts on the prevalence of RDS are scarce. As a result, the primary goal of this study was to determine the prevalence and risk factors for respiratory distress syndrome in neonates admitted to Gurage zone hospitals' neonatal intensive care units. The findings of this study will be important in developing strategies to reduce morbidity and mortality associated with neonatal respiratory distress.

## Method and materials

### Study design

We conducted a retrospective cross-sectional study to assess the prevalence and associated factors of RDS among neonates admitted to neonatal intensive care units in Gurage zone hospitals.

### Study setting and Period

We conducted it at Gunchire primary hospital & Wolkite University specialized hospital. Gunchire's primary hospital is located in Gunchire town, one of the towns in the Gurage zone & which is around 192 km from Addis Ababa and 301 km from Hawassa, the administrative capital of the southern nation and nationality and peoples of Ethiopia. The Wolkite University specialized and teaching hospital is located in Wolkite town, located 150 km from Addis Ababa.

### Study participants and eligibility

All neonates admitted to NICU in Gunchire primary and WKUS hospitals in the previous two consecutive years (from June 2019 to June 2021) were included in the study and Incomplete neonates' medical cards were excluded.

### Sample size determinations

The sample size was determined using a single population proportion using the proportion of RDS 42.9% 6. By assuming, Z α/2=critical value for normal distribution at 95% confidence level, 5% margin of error (W). After assuming a 5% non-response rate the final sample size was 395.



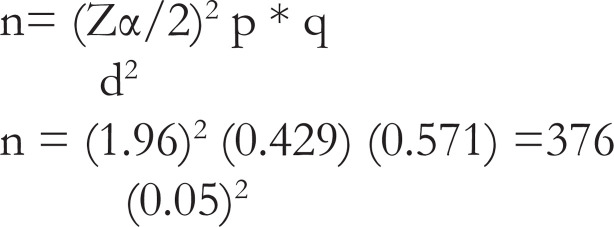



After adding a 5% non-response rate the sample size was 395.

### Sampling technique and procedure

Wolkite University specialized and Gunchrie hospitals each have their own organized NICU, with an average of 1788 admissions to the NICU every two years. We selected a total sample size of 395 neonates from the two hospitals. All neonates admitted to the two hospitals during the study period were considered for the study. The participants were then selected using a simple random sampling method.

### Sampling procedure

We used random sampling to select hospitals from all public hospitals in the Gurage zone. After we built the sampling frame, we chose participants who met the inclusion criteria using Excel (computer-generated random sample). We calculated the number of study subjects assigned to each health facility based on the proportion of neonate medical records.

### Data collection tool

Data were collected by reviewing the patients' cards using a pretested checklist. RDS was confirmed by checking the neonate medical charts.

### Data quality control

Designing appropriate data abstraction tools ensured data quality. Experienced researchers evaluated the checklist and pretested it on 5% of the sample size. Both data collectors and supervisors received training on the data abstraction checklist and data collection process. We maintained close supervision and monitoring during data collection.

### Statistical analysis

Data was cleaned, checked for inconsistencies and missed values, coded and entered using epi info, and exported for analysis. We entered the data into EPI data 3.1. It was checked for completeness and consistency before being exported to SPSS version 25. The data were processed by SPSS version 25 to estimate the prevalence of respiratory distress syndrome. The results of the study were presented with tables, figures, and charts.

## Result

### Socio-demographic characteristics of mothers of Neonates

Among 395 neonatal charts reviewed, 377 (95.4%) records met the inclusion criteria & included in the final analysis. Of the three hundred seventy-seven participants, the majority, 248 (65.8%) were male. Most of the mothers, 316(83.8%) were between 20-34 years of age & the majority (71.1%) of the neonates were from the rural area. (Table 1)

**Table 1:** Socio-demographic characteristics of mother at Gunchire primary hospital and Wolkite University specialized hospital, Gurage zone, South Ethiopia, August 2021(n=377)

Variables	Categories	Frequency	Percentage (%)
Age of mother	<20	21	5.6
	20-35	317	84.1
	>=35	39	10.3
Place of residence	Rural	268	71.1
	Urban	109	28.9
Age in hrs at admission	<24	215	57
	24-72	83	22
	>72	79	21
Sex	Male	247	65.8
	Female	128	34.2
Gestational age in weeks	<35	199	52.8
	35-37	17	4.5
	37-42	148	39.3
	>42	13	3.5

### Obstetric history of mothers at Gunchire primary and Wolkite University Specialized Hospitals

For most of the mothers, 201(53.3%) were multiparous, and based on gestational age, 241(63.9%) women attended antenatal appointments& most of the neonates, 198(52.5%) were <37 weeks. (Table 2).

**Table 2:** Obstetrics history of the mothers of neonates at the Gunchire primary hospital and Wolkite University specialized hospital, Gurage zone, South Ethiopia, August 2021(n=377)

Variables	Categories	Frequency	percentage
Parity	Primi-parous	176	46.7
	Multiparous	201	53.3
Did the mother have an	Yes	241	63.9
ANC follow up?	No	136	36.1
Types of pregnancy	Single	268	71.1
	Multiple	109	28.9
Mode of delivery n=375	Spontaneous vaginal delivery	206	54.9
	Caesarean section	109	29.1
	Instrumental	60	16
Setting of delivery	Hospital	253	67.1
	Health Center	58	15.4
	Private health facility	32	8.5
	Homes	34	9
Obstetrics complication	Yes	165	43.8
	No	212	56.2
Mothers' medical problem	Yes	110	29.2
	No	267	70.8
Chronic hypertension	Yes	69	18.3
	No	308	81.7
HIV	Yes	7	1.9
	No	370	98.1
Diabetes mellitus	Yes	42	11.1
	No	335	88.9
Anaemia	Yes	21	5.6
	No	356	94.4
Other	Yes	48	12.7
	No	329	87.3

### Neonatal characteristics

Among neonates included in the study, 170 (45.1%; 95% CI: 40-50.3%) had RDS. More than two-thirds, 253(67.1%) neonates were delivered to the hospital. For most of the neonates, 260(69%) first-minute APGAR score was <7(Table 3).

**Table 3:** Neonatal characteristics at the Gunchire primary hospital and Wolkite University specialized hospital, Gurage zone, South Ethiopia, August 2021(n=377)

Variable	Categories	Frequency	Percentage (%)
Birth weight in grams	<2500	82	21.8
	2500-4000	292	77.5
	>4000	3	0.8
	No	217	83.8
Sepsis	Yes	228	60.5
	No	149	39.5
Other	Yes	51	13.5
	No	326	86.5
APGAR 1stminute	<7	260	69
	>or=7	97	25.7
APGAR 5^th^ minute	<7	171	45.4
	>or=7	186	49.3
Length of hospital stay in days	0-7	295	78.2
	>8	82	21.8
Status of neonate	Dead	87	23.1
	Alive	277	73.5
	Referred	13	3.4

### Factors associated with respiratory distress syndrome

In the multivariable analysis, male gender, Jaundice, maternal age <20 years&>=35 years, neonatal sepsis, and gestational age of between 35 &37 weeks gestation and mode of delivery were associated significantly with higher rates of RDS. The odds of RDS in male neonates were 1.51 times higher than their counterparts {AOR: 1.51 (0.93, 2.45)}. The odds of RDS in neonates from mothers with gestational age between 35 &37 were 3.99 times higher compared to term gestation {AOR: 3.99 (1.26, 12.62)}. This study also indicated that neonatal jaundice and sepsis {AOR: 4.33 (1.68, 11.17)} and {AOR: 1.92 (1.16, 3.17)} were associated with higher rates of RDS as compared with non-jaundiced and non-sepsis neonates respectively. The odds of RDS in neonates born via Caesarean section were 1.7 times higher than those with spontaneous and instrumental delivery {AOR: 1.70 (1.03, 2.81)}. RDS was higher in neonates from mothers <20 years of age & >=35 years old {AOR: 4.95 (1.69, 14.35)} and {AOR: 5.2, (2.27, 11.88) respectively (Table 4).

**Table 4:** Bivariable and multivariable logistic regression among neonates attending NICU of Gunchire primary hospital and Wolkite University specialized hospital, Gurage zone, South Ethiopia, August 2021(n=377)

Variables	Categories	RDS		COR (95%CI)	AOR (95%)	p-value
		Yes	No			
Age of mother	<20	15(71.4%)	6(28.6%)	3.79(1.43, 10.03)	4.95(1.69, 14.35)	0.003
	20-35	126(39.7%)	191(60.3%)	1	1	
	>=35	29(74.4%)	10(25.6%)	4.4(2.07, 9.33)	5.2(2.27, 11.88)	<0.0001
Place of	Rural	114(42.5%)	154(57.5%)	0.70(.45, 1.09)	0.94(.57, 1.57)	0.814
residence	Urban	56(51.4%)	53(48.6%)	1	1	
Mode of	Caesarean	58(52.3%)	53(47.7%)	1.50(.96, 2.35)	1.7(1.03, 2.81)	<0.039
delivery	section					
	Spontaneous	112(42.1%)	154(57.9%)	1	1	
	&Instrumental					
Age in Hrs	<24	105(48.8%)	110(51.2%)	1.48(.87, 2.5)	1.26(.71, 2.23)	0423
	24-72	48(57.8%)	35(42.2%)	2.12 (1.13, 3.98)	1.491(.75, 2.96)	0.256
	>72	31(39.2%)	48(60.8)	1	1	
Sex of neonate	Male	119(48%)	129(52%)	1.41(0.91, 2.17)	1.51(0.93 2.45)	0.01
	Female	51(39.5%)	78(60.5%)	1	1	
Gestational age	<35	92(46.2%)	107(53.8%)	1.23(0.8, 1.88)	1.33(.83, 2.13)	0.234
	35-37	12(70.6%)	5(29.4)	3.42(1.15, 10.21)	3.99(1.26, 12.62)	0.019
	37-42	61(41.2%)	87(58.8%)	1	1	
	>42	5(38.5)	8(61.5%)	0.89(0.288, 2.85)	0.89(.24, 3.25)	0.859
Jaundice	Yes	196(56.5 %)	151(43.5%)	2.41 (1.04, 5.55)	4.33(1.68, 11.17)	0.002
	No	22(73.3%)	8(26.7%)	1	1	
Hypothermia	Yes	82(51.3%)	78(48.7%)	1.54(1.02, 2.33)	1.2 (.76, 1.90)	0.434
	No	88(40.5%)	129(59.5%)	1	1	
	Yes	87(58.4%)	62(41.6%)	1.42(.94, 2.14)	1.92(1.16, 3.17)	0.011
Sepsis	No	131(57.5%)	97(42.5%)	1	1	

## Discussion

The prevalence of neonatal respiratory distress syndrome was 45.1% (95% CI:40.3%- 50.3). This figure was consistent with the study conducted in Egypt(45.8%)^7^, Black Lion Specialized Hospital, Addis Ababa42.9%^2^. This finding is in line with a study in China^8^. The finding is higher than studies conducted in several countries including Nepal(34%)^9^ ,India (33.4%)^10^, Pakistan (4.6%)^11^, and Portugal(8.83%)^12^, Nigeria(26.2%) 13/a>), Iraq(34.7%) 14, Jimma Ethiopia(11.9%)^15^. But, the magnitude was lower than studies from Saudi Arabia^16^, Cameroon (47.5%)^17^, and Poland (54.29%)^18^, North India 57% 19. This difference may be due to health service coverage, health service quality, font-weight: normal”>research study design, and population understanding of health service.

In this study, we identified multiple factors of RDS in neonates, including Apgar score<7, sepsis, Jaundice, maternal Age <20, and sex of the neonate (male). Apgar score of <7 during the first minutes of delivery was a significant factor & supported by a study conducted in black lion hospital Addis Ababa^6^.

Being male was a significant factor in developing RDS, which is consistent with studies conducted in China^12^, Cameron^17^, Addis Ababa^6^ Ethiopia)^5^, the United States^20^, and South Korea^21^. Deaths secondary to RDS are consistently higher in male neonates 22, and Bayi Children's Hospital, China^23^. This aligns with the fact that male neonates have higher levels of circulating testosterone, which may lead the differences in pulmonary biomechanics and vascular development.

Caesarian sections were associated with an increased risk of neonatal RDS in the meta-analysis study^8^, ^20^, ^21^ & at Zhengzhou University hospital and Bayi Children's Hospital, China^23^, ^24^.

Being gestational age between 35 &37 weeks was a significant factor. This finding is related to evidence from a retrospective study on the risk of respiratory distress syndrome in singleton pregnancies with preterm premature rupture of Membranes between 24+0 and 36+6 Weeks in Poland^18^, Bayi Children's Hospital, China^23^

Qena University Hospital(Egypt), which prematurity and maternal diabetes were the most risk factors associated with respiratory diseases^25^, and in Ethiopia 26 But, Besides early preterm infants, full-term and late preterm have a growing trend in the pathogenesis of neonatal respiratory distress syndrome if the delivery was via cesarean section 27, and incidence of RDS in neonates were the same at different gestational age^28^.

Jaundice was significantly associated with RDS. Neonatal sepsis was significantly associated with RDS as was evidenced by other studies in Egypt^29^, Nepal^9^, and Ethiopia^26^. Maternal age < 20 years and >=35 years old was found to be a significant factor in developing RDS. But, Maternal age was not associated with an increased risk of respiratory syndrome in other studies, Italia^28^. This present study does have some limitations. As the data were collected from medical records, socioeconomic factors and some basic information were not possible to collect for the whole cohort, as they might be associated with respiratory distress syndrome; therefore, this variable was not included in analyses.

## Conclusion and recommendation

The prevalence of RDS in the study area was high and is continuing to be a major public health problem in neonates admitted to NICU. Being male, Jaundice, maternal age <20 and>=35 years, neonatal sepsis, and mode of delivery were statistically significant factors of RDS. Thus, to reduce the burden of RDS in neonates, Health care providers should give special emphasis on neonates delivered via Caesarean section and if they are from young and advanced maternal age.

## Acronyms and abbreviation

GA: Gestational age
GPH: Gunchire primary hospital
NRDS: Neonatal respiratory distress syndrome
RDS: Respiratory distress syndrome
WUSH: Wolkite university specialized hospital

## Availability of data materials

The datasets analysed during the current study are available from the corresponding author upon reasonable request and will be attached to the editorial office when requested.
